# Involvement of the different lung compartments in the pathogenesis of pH1N1 influenza virus infection in ferrets

**DOI:** 10.1186/s13567-016-0395-0

**Published:** 2016-11-08

**Authors:** Beatriz Vidaña, Jorge Martínez, Jaime Martorell, María Montoya, Lorena Córdoba, Mónica Pérez, Natàlia Majó

**Affiliations:** 1 Departament de Sanitat i Anatomia Animals, Universitat Autònoma de Barcelona, Bellaterra, Barcelona, Spain; 2 IRTA, Centre de Recerca en Sanitat Animal (CReSA, IRTA-UAB), Campus de la Universitat Autònoma de Barcelona, Bellaterra, Barcelona, Spain; 3 UAB, Centre de Recerca en Sanitat Animal (CReSA, IRTA-UAB), Campus de la Universitat Autònoma de Barcelona, Bellaterra, Barcelona, Spain; 4 Departament de Medicina i Cirurgia Animals, Universitat Autònoma de Barcelona, Bellaterra, Barcelona, Spain

## Abstract

Severe cases after pH1N1 infection are consequence of interstitial pneumonia triggered by alveolar viral replication and an exacerbated host immune response, characterized by the up-regulation of pro-inflammatory cytokines and the influx of inflammatory leukocytes to the lungs. Different lung cell populations have been suggested as culprits in the unregulated innate immune responses observed in these cases. This study aims to clarify this question by studying the different induction of innate immune molecules by the distinct lung anatomic compartments (vascular, alveolar and bronchiolar) of ferrets intratracheally infected with a human pH1N1 viral isolate, by means of laser microdissection techniques. The obtained results were then analysed in relation to viral quantification in the different anatomic areas and the histopathological lesions observed. More severe lung lesions were observed at 24 h post infection (hpi) correlating with viral antigen detection in bronchiolar and alveolar epithelial cells. However, high levels of viral RNA were detected in all anatomic compartments throughout infection. Bronchiolar areas were the first source of IFN-α and most pro-inflammatory cytokines, through the activation of RIG-I. In contrast, vascular areas contributed with the highest induction of CCL2 and other pro-inflammatory cytokines, through the activation of TLR3.

**Electronic supplementary material:**

The online version of this article (doi:10.1186/s13567-016-0395-0) contains supplementary material, which is available to authorized users.

## Introduction

In 2009, the swine-origin H1N1 influenza A virus (IAV) emerged and caused outbreaks of respiratory illness in humans around the world. After the 2009 pandemic, outbreaks of that strain have continued to cause serious illness and increased mortality, particularly in young adults and children [1]. The physiopathology of pandemic H1N1 (pH1N1) infection differs between individuals. Whilst most patients develop mild upper respiratory-tract infection [2], some patients progress to develop severe lower respiratory tract complications [3]. In addition, high rates of clinically unapparent infections have been reported by seroepidemiological studies [4, 5]. This evidence suggests that the severity of influenza is, at least, partially determined by host factors.

Severe cases after pH1N1 infection are consequence of diffuse alveolar damage (DAD) [3], also termed interstitial pneumonia, triggered by the spread of pH1N1 infection from the upper to the lower respiratory tract [6, 7] and an exacerbated inflammatory host immune response [8–12]. Several hypotheses have been made about the mechanisms involved in the dysregulation of the host inflammatory response after pH1N1 and IAV infection. However, all of them involve the up-regulation of pro-inflammatory cytokines [13, 14] and the influx of inflammatory leukocytes to the lungs [9, 15].

While leukocytes have traditionally been considered the major source of pro-inflammatory cytokines, there is also a consensus calling attention to the role of epithelial and endothelial cells as important sources of pro-inflammatory cytokines during various infectious processes [16–18]. In a recent study, Brandes et al. [9] showed that lethality in a murine model of acute influenza arose directly from the damage caused by constrained innate inflammation primarily involving neutrophils co-acting with monocytes. However, the first influx of neutrophiles and monocytes needs to be induced by the release of innate immune mediators from lung resident cell populations (respiratory epithelium, endothelium and alveolar macrophages), which first come in contact with the virus.

During pH1N1 infection, leukocyte influx into the lungs is regulated by the signalling effects of cytokines and chemokines released by different lung cell populations in response to infection [13, 14, 19]. Although cytokines have specific functions and are released in a cell-type-dependent manner, all of them are produced/activated via common mechanisms involving the activation of pattern recognition receptors (PRRs) [20, 21]. By these means, several studies have implicated either lung endothelial or epithelial cells as key regulators in the initial release of pro-inflammatory cytokines after pH1N1 infection [10, 14, 16]. However, their relative contribution to cytokine release regulation mechanisms and the dynamics of their release have not entirely been elucidated.

Understanding which cellular types and inflammatory dynamics are involved in the development of the detrimental inflammatory response after IAV infection, is important for the future development of therapeutic strategies, designed to the control of the host immune response. In this way, the modulation of the host immune response will have the potential advantage of exerting less selective pressure on viral populations, an important factor in the development of IAV infection therapies. With the aim to unravel the mechanisms involved in the regulation of the innate immune response against IAV infection, and the relative contribution of the different lung compartments, we investigated the early induction of innate immune molecules, observed in different lung compartments. We tried to discern the cytokine production of the main cell type present in each lung compartment (endothelial cells, epithelial bronchiolar cells and alveolar epithelial cells) by means of a laser capture microdissection (LCM) technique. As the limitations of the technique did not allow us to completely individualize the target cells, we assessed the cytokine expression associated to the viral replication in the different lung compartments (vascular, alveolar and bronchiolar).

## Materials and methods

### Virus

The human pH1N1 isolate A/CastillaLaMancha/RR5911/2009 was used in the present study. The virus was isolated at the National Influenza Centre, Centro Nacional de Microbiología, Instituto de Salud Carlos III (CNM, ISCIII) from a respiratory sample sent by the Spanish Influenza Surveillance System for virological characterization. It was isolated from a 35-years old woman, without co-morbidities, who developed a severe disease and died. The viral isolate was passaged in Madin-Darby Canine kidney (MDCK) cell cultures four times and had a final titre of 10^6.02^ TCID_50_/mL. Titre was determined using the Reed and Muench method [22].

### Ferrets

Eleven neutered male ferrets between 8 and 9 months of age, and seronegative for IAV (influenza A antibody competition multi-species ELISA, ID Screen^®^, France), were randomly selected from a stable, purposely bred colony (*Euroferret*, Denmark). Upon arrival at the Centre de Recerca en Sanitat Animal (CReSA), the animals were placed in biosafety level 3 (BSL-3) facilities. Ferrets were randomly assigned to different experimental groups, separated into experimental isolation rooms and then acclimated during 1 week. The animals were inhabited in standard housing cages for laboratory ferrets (F-SUITE Ferret Housing, Tecniplast, Italy) and they were provided with commercial food pellets and tap water ad libitum throughout the experiment. All experiments were performed under a protocol (no. 1976) that was reviewed and approved by the Animal and Human Experimentation Ethics Commission of the *Universitat Autònoma de Barcelona*. Animals were divided into two groups. The control group included two ferrets and the infected group included nine ferrets which were intratracheally inoculated with 10^5^ TCID_50_/mL of the A/CastillaLaMancha/RR5911/2009 virus diluted in 0.5 mL of phosphate buffer saline (PBS). During inoculation, animals were deep sedated with butorphanol (0.05 mg/kg) (Torbugesic^®^ Vet, S.A., Spain) and medetomidine (0.05 mg/kg) (Domtor^®^ Pfizer, S.A., Spain) administered subcutaneously. After inoculation, animals were reverted from sedation with atipamezole (0.25 mg/kg) subcutaneously administered and monitored by a specialized veterinarian until complete recovering from sedation. Control and infected animals were euthanized at 0 and 12, 24 and 72 h post infection (hpi) respectively. Early times post infection were selected in order to evaluate the innate immune response and assess early immune viral recognition and induction of pro-inflammatory cytokines and chemokines throughout infection. Animals were euthanized by an intravenous injection of sodium pentobarbital (100 mg/kg), under anaesthesia with ketamine (5–10 mg/kg) (Imalgene 1000^®^ Merial, S.A., Spain) and medetomidine (0.05 mg/kg) (Domtor^®^ Pfizer, S.A., Spain), administered subcutaneously. Necropsies were performed in control and infected animals after euthanasia according to a standard protocol.

### Histopathology

Right lung lobe sections (cranial and caudal lobes), were taken for histological examination. The tissues were fixed for 24–48 h in neutral-buffered 10% formalin, and then embedded in paraffin wax in two different blocs containing one portion of the cranial and the caudal right lung lobes, consecutively taken. One of the paraffin blocks was sectioned at 3 µm, and stained with haematoxylin and eosin (HE) for examination under light microscopy; the second paraffin block was used for microdissection studies.

Cross sections of the cranial and caudal pulmonary lobes for each animal were histopathologically and separately evaluated. Semiquantitative assessment of IAV-associated microscopic lesions in the lungs was performed. The lesional scoring was graded on the basis of lesion severity as follows: grade 0 (no histopathological lesions observed), grade 1 (mild to moderate necrotizing bronchiolitis), grade 2 (bronchointerstitial pneumonia characterised by necrotizing bronchiolitis and alveolar damage in adjacent alveoli), and grade 3 (necrotizing bronchiolitis and diffuse alveolar damage in the majority of the pulmonary parenchyma). Microscopic lesional scores were assigned for each lobe, and the means of the two lobes were used for the final histopathological score for each animal.

### Laser capture microdissection

Lung samples from infected and control animals were used for the microdissection study. For each animal, 20 μm sections were cut from formalin-fixed paraffin-embedded (FFPE) lung tissue and mounted in PEN-membrane slides (two sections per slide). Prior to deparaffinization, slides were placed into an oven at 60 °C for 25 min. For each lung sample, sections were cut, deparaffinised, and rehydrated using standard protocols with RNase-free reagents, and stained with 1% cresyl violet acetate (SIGMA, C5042), and alcoholic 1% eosin (Alvarez, 10-3051). Stained slides were then dehydrated through a series of graded ethanol steps prepared with DEPC treated water (Ambion, P/N AM9915G) to 100% ethanol. The slides were then air-dried for 10 min, and individually frozen at −80 °C in 50 mL parafilm sealed falcons, before being transferred to the LCM microscope (LMD6500; Leica Microsystems) for simple microdissection.

**Figure 1:**
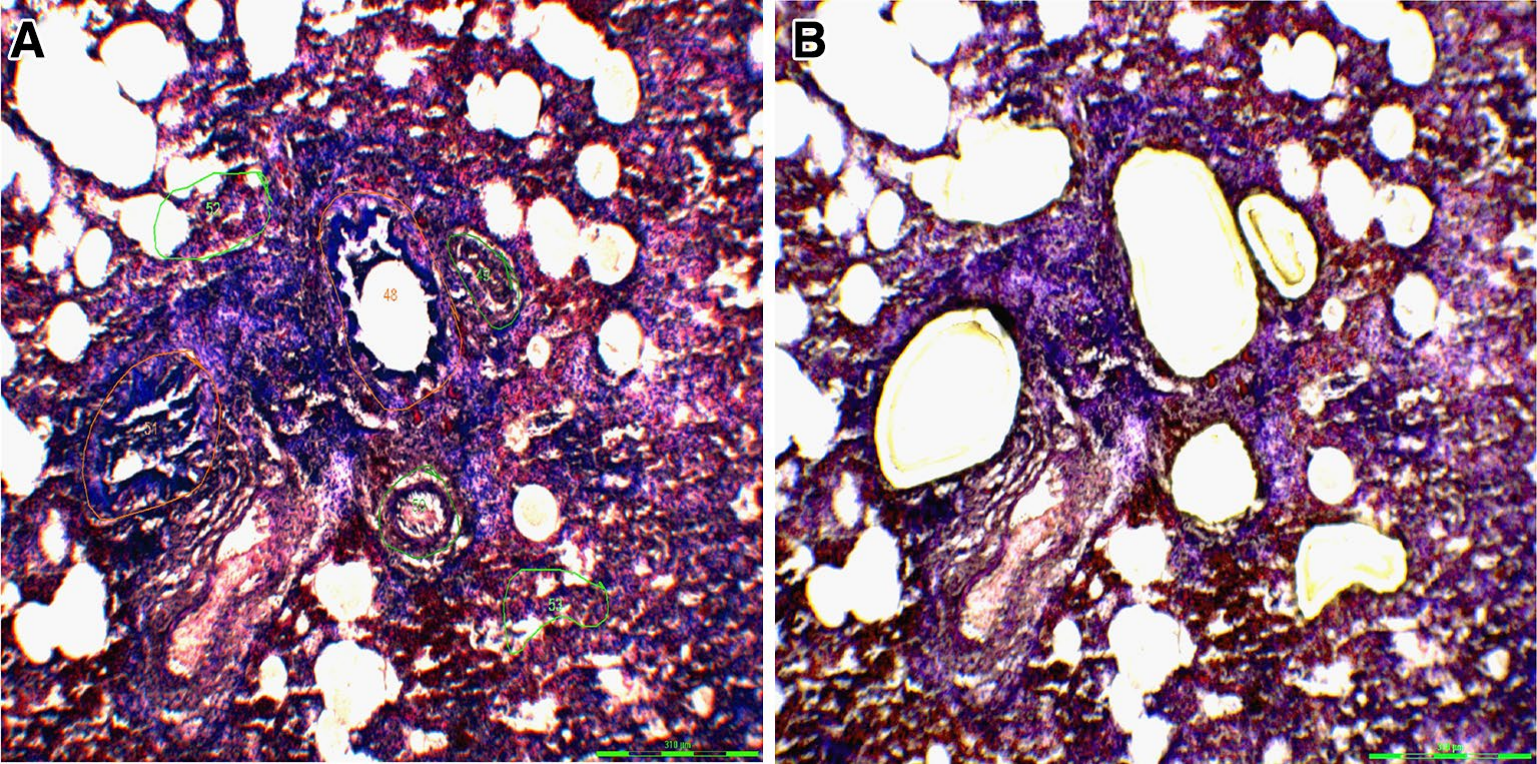
**Laser microdissected lung areas of a pH1N1 infected ferret, at 3 dpi. A** Before laser microdissection, **B** after laser microdissection.

Bronchiolar, vascular, and alveolar areas were separately selected for analysis using the Leica LMD6500 (Leica) system (20× magnification, Laser Microdissection 6000 software version 6.7.0.3754). Selected areas were chosen by a pathologist as observed in Figure 1. In infected animals, areas which exhibited pathological lesions were preferably selected. The total dissected area per selected lung compartment and animal rose approximately to 1.5 mm^2^. One cap was used per anatomic dissected compartment and animal.

The different dissected areas were then collected separately into RNAse-free 1.5 mL PCR tubes, per lung compartment.

### Viral detection and quantification

Viral titration from lungs samples and nasal swabs was performed on control and infected animals, and determined by plaque forming units (PFU) as described previously [23]. A portion of around 0.2 g of the right caudal lung lobe and nasal swabs were placed in 0.5 mL of Dulbecco’s Modified Eagle’s Medium (DMEM) (BioWhittaker^®^, Lonza, Verviers, Belgium) with 600 µg/mL penicillin and streptomycin, and frozen at −80 °C until further processing.

For the detection of IAV antigen by immunohistochemistry (IHC), paraffin sections were stained with a primary antibody against the influenza A nucleoprotein (NP), as previously described [8, 24]. Briefly, an antigen retrieval step was performed using protease XIV (Sigma-Aldrich, USA) for 10 min at 37 °C, and then blocked for 1 h with 2% bovine serum albumin (BSA) (85040C, Sigma-Aldrich Química, S.A., Spain) at room temperature (RT). Samples were then incubated with a commercially available mouse-derived monoclonal antibody (ATCC, HB-65, H16L-10-4R5) concentration (343 mg/mL), as a primary antibody, at a dilution of 1:100 at 4 °C overnight, followed by 1 h incubation with biotinylated goat antimouse IgG secondary antibody (Dako, immunoglobulins As, Denmark). Finally, an ABC system (Thermo Fisher Scientific, Rockjord, IL, USA) was used and the antigen–antibody reaction was visualized with 3, 3′-diaminobenzidine DAB as chromogen. Sections were counterstained with Mayer’s haematoxylin. The positive control consisted of a FFPE lung from a ferret and of a FFPE heart from a chicken, experimentally infected with influenza. The same sections in which the specific primary antibody was substituted with PBS or an irrelevant antibody [anti-Porcine Circovirus type 2 (PCV2), diluted 1:1000] were used as negative controls. Semiquantitative assessments of IAV antigen expression in the lungs were performed. The positive cells in six arbitrarily chosen 20× objective fields in alveolar areas, and five arbitrarily chosen 20× objective fields in bronchial or bronchiolar areas, were quantified separately in each lung lobe (cranial and caudal) for every animal. The mean of the total cell counts per field across two lobes was calculated for each animal.

Viral RNA from total lung samples (proximal mediastinic right caudal lobe section of 5 mm^2^) was extracted with NucleoSpin^®^ RNA Virus Kit (Macherey–Nagel, Düren, Germany), following the manufacturer’s instructions. IAV matrix (M) gene was then quantified from the RNA extracted by the Taq-Man one-step quantitative real time PCR (RRT-PCR) using the primers and probe described in Additional file 1 [25]. One-step RRT-PCR master mix reagents (Applied Biosystems, Foster City, CA, USA) were used following the manufacturer’s instructions using 5 μL of eluted RNA in a total volume of 25 μL. The amplification conditions were as follows: reverse transcription at 48 °C for 30 min; initial denaturation reaction at 95 °C for 15 min and 40 PCR-cycles of 95 °C 15 s, and 60 °C 1 min. Reaction was performed using Fast7500 equipment (Applied Biosystems). Samples with a Ct value ≤40 were considered positive for influenza viral RNA detection.

Viral RNA detection from different lung anatomic compartments was extracted using the miRNeasy FFPE Kit (no. 217504, Qiagen, Valencia, CA, USA) and the RNA stabilisation and on-column DNase digestion protocols (Qiagen), following the manufacturer’s instructions. Briefly, the transfer film with the attached dissected material, per animal and lung compartment, was placed in deparaffinization melting buffer at 72 °C for 10 min, and then treated with Proteinase K at 60 °C for 45 min. Isolated RNA was concentrated using ethanol precipitation method and membrane column attachment. RNA was eluted twice in 14 μL total DEPC water, yielding between approximately 5 and 9 ng/μL of RNA, per sample. RNA extracts were stored at −80 °C until required. Reverse transcription was performed using an ImProm-II reverse transcription system, with random primers (Promega, Madison, WI, USA), using 10 µL of the eluted RNA. IAV M gene was then quantified on the RNA, extracted by two-step RRT-PCR using the forward and reverse primers for detection of the M gene, described in Additional file 1. Briefly, RRT-PCR was performed using a Power SYBR green kit, (Applied Biosystems) and Fast 7500 equipment (Applied Biosystems). PCR reactions were performed in 10 μL reaction volumes, in quadruplicates; 45 amplification cycles were used, and the annealing temperature was 60 °C. Results are expressed as inverted Ct values. Samples with a Ct value ≤45 were considered positive for influenza viral RNA detection.

### Gene expression profiles

Total RNA was isolated from LCM tissues, using the miRNeasy FFPE Kit (no. 217504, Qiagen), as described in the above section.

Relative mRNA expression levels of IFNα, IL-6, TLR-3, IL-8, RIG-I, IFNγ, TNFα, CCL2, CCL3 and the housekeeping gene β-actin, at each different lung compartment, were assessed by two-step RRT-PCR. Primer sequences and source are illustrated in the Additional file 1. Amplicon sizes of the target genes range between 90 and 120 base pairs. Briefly, RRT-PCR was performed using a Power SYBR green kit (Applied Biosystems) and Fast 7500 equipment (Applied Biosystems). RNA extraction was performed on the ferret lung tissue samples with an RNeasy Mini Kit (Qiagen), using the RNA stabilization and on-column DNase digestion protocols (Qiagen). Reverse transcription was performed using an ImProm-II reverse transcription system (Promega, Madison, WI, USA), at 0.5 µg RNA. PCR reactions were performed in 10 µL reaction volumes in quadruplicates; 45 amplification cycles were used, and the annealing temperature was 60 °C. The expression levels were normalized using the house-keeping gene β-actin using the relative standard curve method and taking into account primer efficiency. The results are expressed as arbitrary units.

### Vascular gene expression

Relative mRNA expression levels of selectin P-ligand (SELPLG), and the housekeeping gene β-actin in the vascular compartment, were assessed by two-step RRT-PCR, as described in the above section. Briefly, RRT-PCR was performed using a Power SYBR green kit (Applied Biosystems) and Fast 7500 equipment (Applied Biosystems). PCR reactions were performed as described in the above section and primer sequences and the GenBank accession number are illustrated in Additional file 1. The expression levels were normalized using the house-keeping gene β-actin using the relative standard curve method and taking into account primer efficiency. The results are expressed as arbitrary units. Primer sequences for the SELPLG gene was designed as described previously [26]. The ferret-specific SELPLG gene is available in the NCBI nucleotide database [27]. The amplification product was detected by electrophoresis to validate the size of the product, in accordance with the primer design, and the products were purified using a QIAquick PCR Purification Kit (Qiagen). Sequencing reactions were performed with ABI Prism BigDye Terminator Cycle Sequencing v.3.1 Ready Reaction (Applied Biosystems), and analysed using an ABI PRISM model 3730 automated sequencer (Applied Biosystems). The amplified sequence correlated with the ferret specific target sequences.

### Statistical analysis

Data visualization was performed with GraphPad Prism 6 (GraphPad Software, La Jolla, CA, USA). All statistical analysis was performed using SPSS 15.0 software (SPSS Inc., Chicago, IL, USA). For all analysis, ferret was used as the experimental unit. The significance level (α) was set at 0.05. The Shapiro–Wilk’s and the Levene test were used to evaluate the normality of the distribution of the examined quantitative variables, and the homogeneity of variances, respectively. No continuous variable that had a normal distribution was detected. Thus, a non-parametric test (Wilcoxon test) using the U Mann–Whitney test was used to compare the different values obtained for all the parameters (histopathology and viral load), between groups (control versus infected), and between different compartments of the infected group (alveolar, bronchiolar and vascular), for viral load and gene expression profiles, at all sampling times.

## Results

### Histopathology

**Figure 2:**
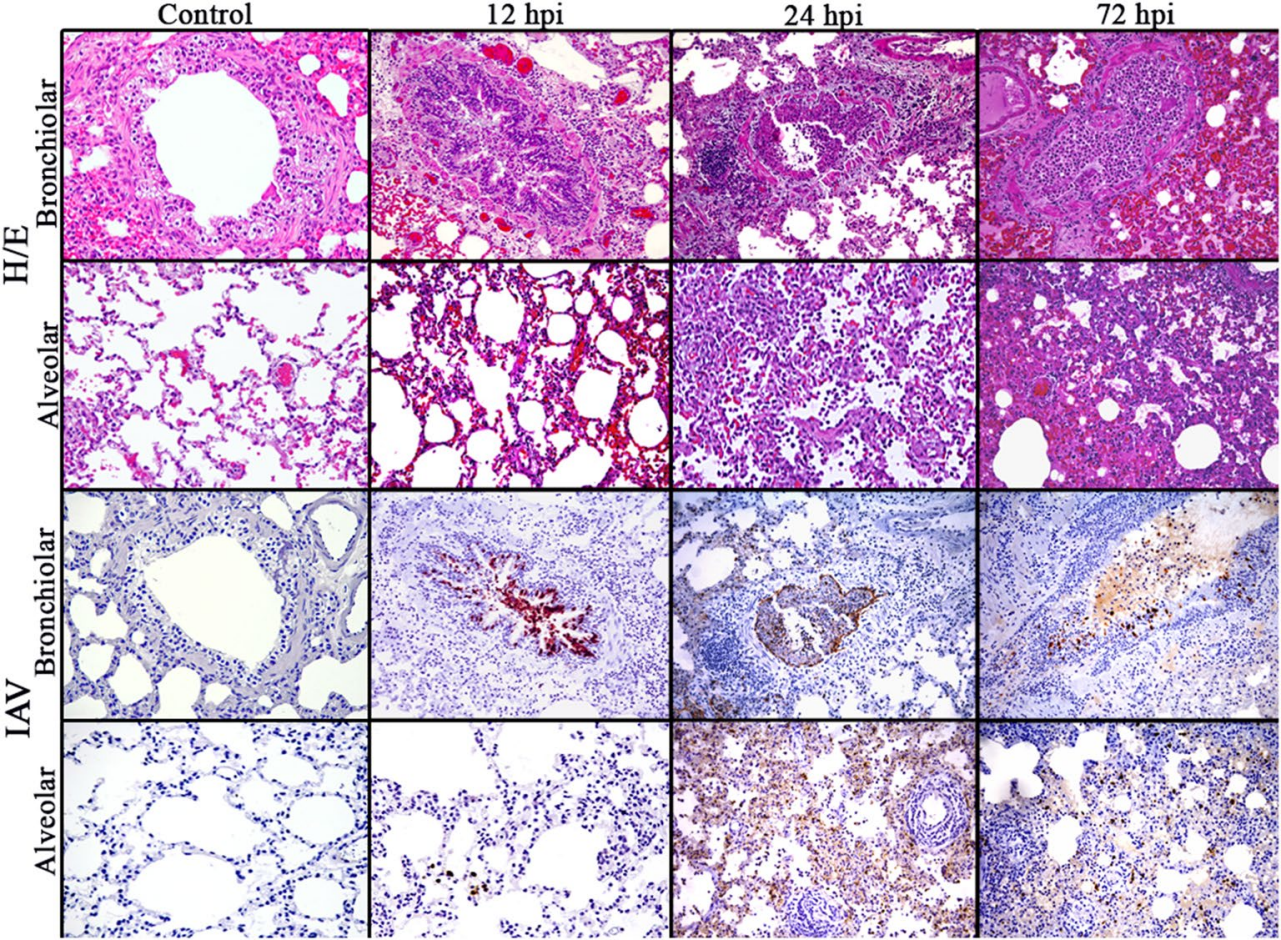
**Pulmonary histopathological lesions in pH1N1-infected ferrets at different times post infection.** Pictures show healthy lung sections, and different severity of lesions in alveolar and bronchiolar areas, and the distribution of viral antigen in the respective areas. Each picture shows the representative histopathological lesions observed in control animals previous to infection, and in infected animals, at each time post infection (three animals evaluated per time-point). HE and immunohistochemical staining for IAV NP antigen (×20 objective field).

Histopathological evaluation identified lesions mainly localized in bronchial/bronchiolar and alveolar areas (Figure 2). At 12 hpi, histopathological lesions were characterised by mild bronchiolitis, consisting of bronchial/bronchiolar epithelium necrosis, and the presence of a mild macrophagic infiltrate in the bronchial/bronchiolar lumen. Lesions were similar in both cranial and caudal pulmonary lobes at this stage. At 24 hpi, lesions were consistent with bronchointerstitial pneumonia, or, interstitial pneumonia in the caudal lobes. Bronchointerstitial pneumonia was characterised by bronchial/bronchiolar necrosis and alveolar epithelial necrosis, lymphoplasmacytic infiltration, with mucus and cell debris filling the bronchioli and the adjacent alveoli. In addition, two of the three animals presented acute interstitial pneumonia (DAD) in caudal lobes, at this time point.

As expected, after infection, inflammation affected firstly the bronchioli (bronchiolitis) at 12 hpi followed by extension to surrounding alveoli (bronchointerstitial pneumonia) at 24 and 72 hpi. However, some animals developed a more extended inflammation of alveolar septa (DAD) at 24 hpi. DAD was characterised by a moderate to severe bronchiolar necrosis with lymphoplasmacytic infiltration in the lamina propria, and a variable number of macrophages and neutrophils in the lumen. Vascular and alveolar oedema was also observed accompanied by inflammatory infiltrates in alveolar and perivascular areas. The alveolar septa were also congested, and presented necrosis of type-1 pneumocytes cells accompanied by interstitial inflammatory infiltrates in the septa and lumen, mainly characterised by macrophages and neutrophils. At 72 hpi, lesions were milder than at 24 hpi and consisted of mild bronchointerstitial pneumonia.

**Figure 3:**
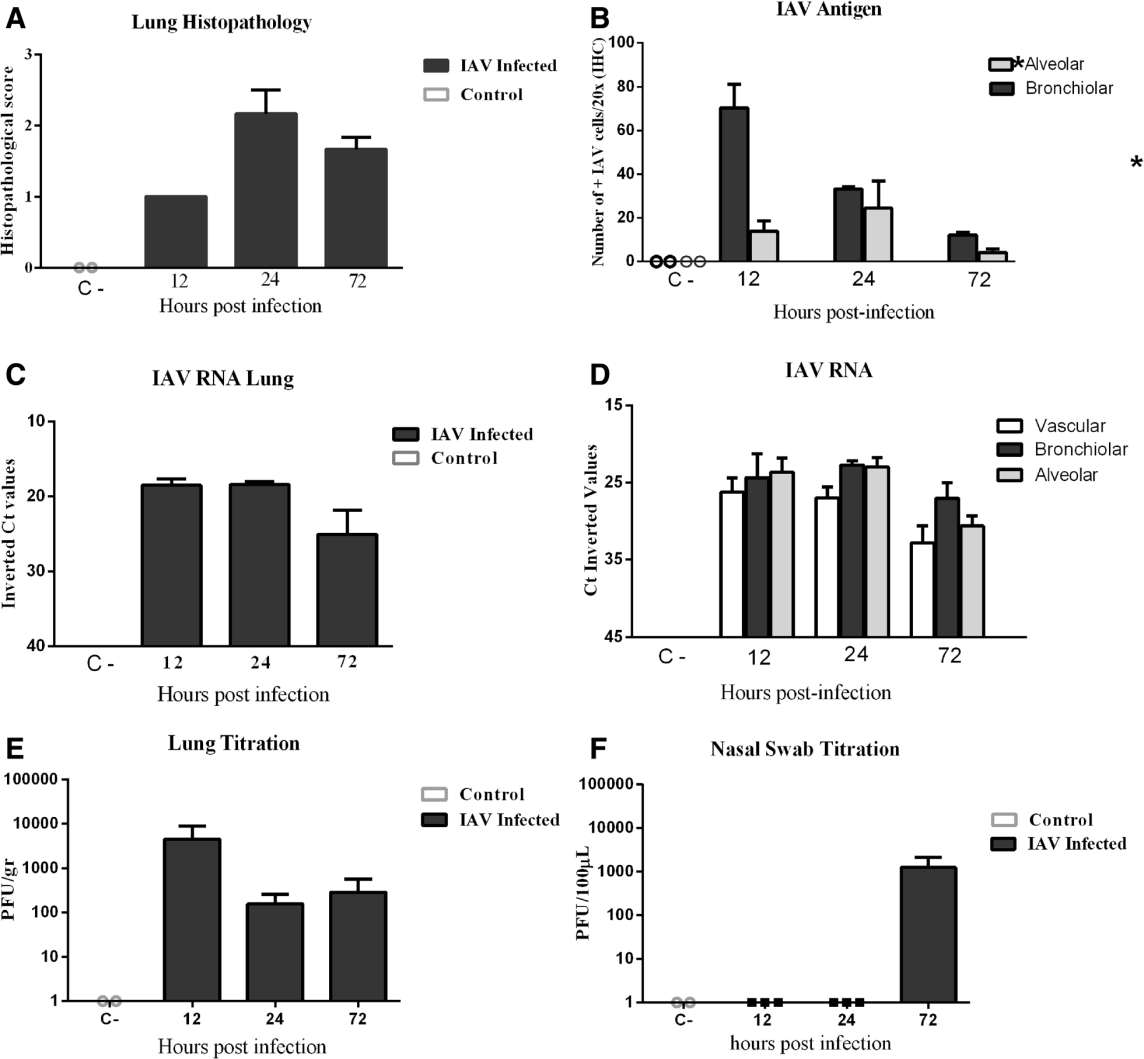
**Histopathological scores, IAV antigen quantification by IHC and viral RNA detection by RRT-PCR in the lungs. A** Histopathological scores representing lung lesion severity in infected and control (C-) animals. **B** Immunohistochemical cell quantification of IAV-positive cells in infected and control ferret lungs; results express the mean cell counts with SEM (×20 objective field). **C** IAV RRT-PCR in the total lung of infected and control animals and **D** in different lung compartments of infected and control animals. Values represented as inverted Ct values. **E** Lung viral titration. Values represented as PFU/g. **F** Nasal swab viral titration. Values represented as PFU/µL. The data expresses the means with SEMS. Statistically significant differences are represented by *p < 0.05.

Higher histopathological scores were observed in the infected group in comparison with the control group, which did not present any histopathological lesion. Of infected animals, lower histopathological scores were observed at 12 hpi, while higher histopathological scores were observed at 24 hpi. At 72 hpi, histopathological scores slightly decreased in comparison to 24 hpi (Figure 3).

### Viral detection and quantification

Viral detection and quantification by IHC was performed in lung tissues. In general, viral antigen was observed in the nucleus of bronchial and bronchiolar epithelial and glandular cells of infected animals at 12 hpi (Figure 2).

At 24 hpi, IAV antigen was observed in the nucleus of the respiratory epithelium (bronchial, bronchiolar and alveolar), and in the nuclei and cytoplasm of macrophages. In the alveolar epithelium, positive reaction was mainly observed in type II pneumocytes, but also in type I pneumocytes (Figure 2).

Quantification of IAV positive cells in the lungs of infected animals revealed that higher numbers of positive cells were observed in bronchiolar areas at 12 hpi. Statistically, significant higher numbers of positive cells in bronchial areas were observed in comparison with alveolar areas at 12 and 72 hpi (Figure 3) (*p* < 0.05).

Viral RNA detection by RRT-PCR in the total lung, and in the different lung compartments, and viral titration by PFU in the total lung was performed. Control animals were negative to viral titration and to viral RNA detection in the total lung, and in the different lung compartments (Figure 3). In the total lung, higher viral RNA detection was observed at 12 and 24 hpi, and then levels decreased at 72 hpi. Viral RNA detection in the different anatomic compartments of infected animals, showed higher viral RNA levels in bronchiolar and alveolar areas at 24 hpi, followed by 12 hpi. Vascular areas showed higher levels of viral RNA in comparison to control animals, at all-time points.

Viral titration in the total lung showed higher viral titers at 12 hpi, which decreased to similar levels at 24 and 72 hpi. Viral secretion on nasal swabs was only observed in animals at 72 hpi (Figure 3).

### Gene expression profiles

**Figure 4:**
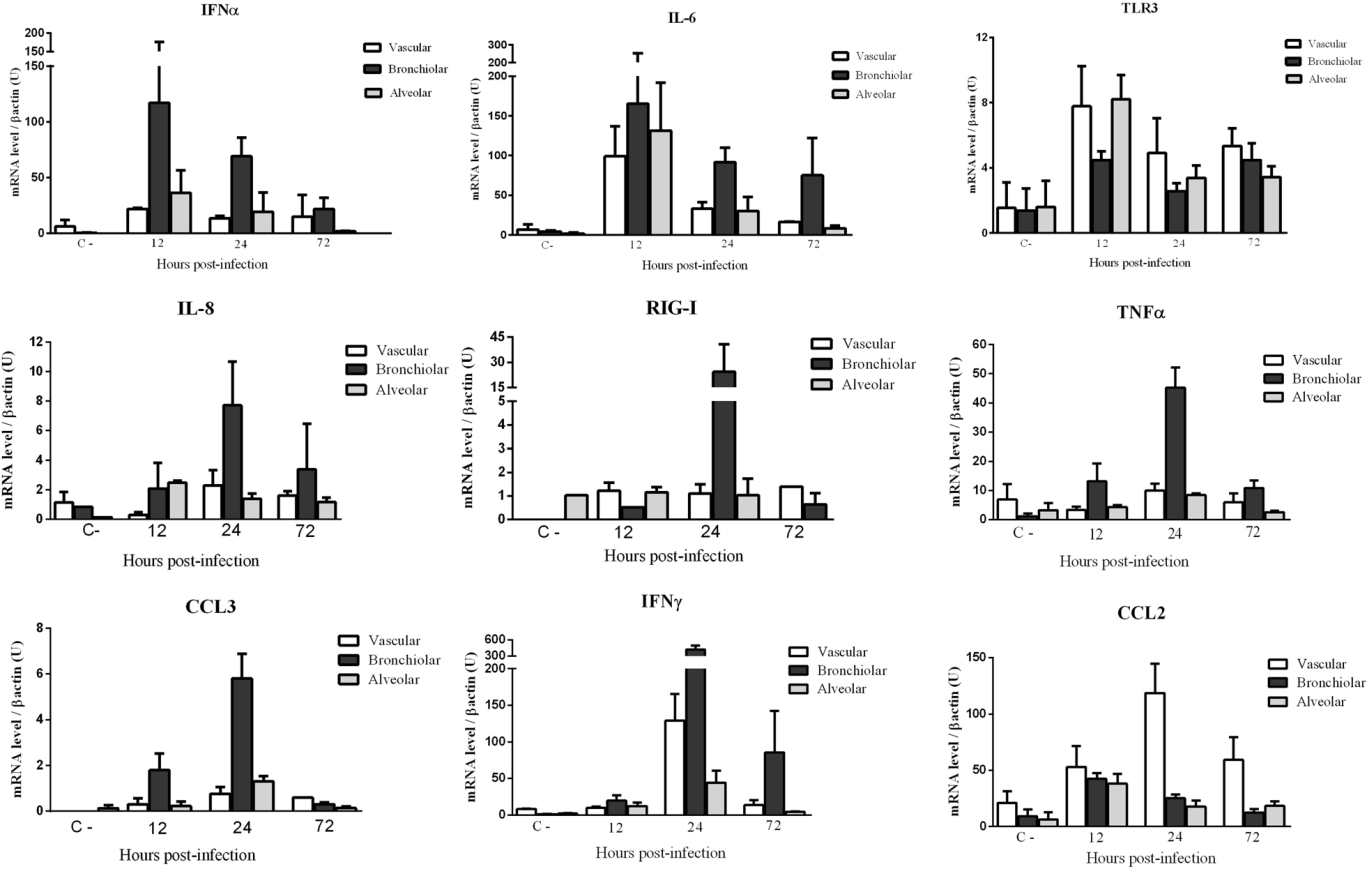
**Innate immune gene expression by RRT-PCR in vascular, bronchiolar, and alveolar areas in the lungs.** Comparisons of the gene expression levels of IFNα, IL-6, TLR3, IL-8, RIG-I, TNFα, CCL3, IFNγ and CCL2, in different anatomic lung compartments of control (C-) and infected ferrets at 12, 24, and 72 hpi. The data expresses the mean with the SEMs. No statistically significant differences were observed between groups.

Innate immune gene expression levels observed in the different anatomic lung compartments are represented in (Figure 4). Innate immune gene expression levels were higher in all anatomical compartments, in comparison with control animals.

Higher levels of IL-6 and TNFα gene expression were observed in bronchiolar areas, in comparison with alveolar and vascular areas along the experimental period. IL-6 gene expression levels were higher at 12 hpi while the highest TNFα levels were detected at 24 hpi.

Higher IL-8, CCL3 and CCL2 gene expression levels were observed at 24 hpi. IL-8 and CCL3 were more expressed in bronchiolar areas, while CCL2 was more expressed in vascular areas. Regarding the IFN response, IFNα gene expression levels were higher in bronchiolar areas at early stages post-infection (12 dpi), while IFNγ was highly expressed also at bronchiolar areas, but in later stages of infection (24 and 72 hpi). Interestingly after bronchiolar expression, high levels of IL-6 and IFNγ were also observed in alveolar and vascular areas at 12 and 24 hpi. Besides, a higher expression of IL-8 in alveolar areas was observed at 12 hpi. With regard to PRRs expression, RIG-I gene was very highly expressed r in bronchiolar areas at 24 hpi, while no major differences in expression were observed in alveolar or vascular areas throughout infection. In contrast, TLR3 was highly expressed in all the compartments, but significantly in alveolar and vascular areas at 24 hpi.

### Vascular gene expression

Vascular adhesion was evaluated by the expression of SELPLG gene in vascular areas. Infected animals showed higher levels of SELPLG gene expression when compared to control animals. SELPLG expression was upregulated throughout the infection (see Additional file 2).

## Discussion

Three main mechanisms are suggested to be involved in pH1N1 pathogenicity: (i) alveolar epithelial necrosis, caused by viral cytopathogenicity, and the release of cytokines by infected cells; (ii) activation of lung vasculature, which increases endothelial permeability, releases cytokines, and triggers leukocyte migration; and (iii) damage triggered by the inflammatory infiltrates, mainly formed by neutrophils and macrophages, plus the release of cytokines from these cells [10]. These mechanisms work in a feed forward manner, eventually increasing the inflammatory damage produced.

In this study, inflammation was firstly observed in bronchiolar compartments, in association with viral replication in bronchiolar epithelial cells and the higher expression of IFNα and IL-6, early after infection. These findings were expected, since the bronchiolar epithelium is one of the first targets after IAV infection [10, 28]. Later, more severe histopathological lesions were observed in correspondence with higher presence of leukocytes and the infection of alveolar epithelial cells. Concurrently, bronchiolar areas presented the higher expression of TNFα, CCL3, IL-8 and IFNγ. Alveolar areas were also shown to contribute to the influx of inflammatory cells by the up-regulation of IFNα, IL-6 and IL-8, at 12 hpi, and CCL3 and IFNγ, at 24 hpi. The up-regulation of IL-8, IFNγ and CCL3 has been related to lung recruitment of neutrophils, NK and T cells and the development of severe influenza infection [8, 14, 19, 29]. In addition, TNFα and IL-6 up-regulation mediate phagocytic inflammatory infiltration and the apoptosis of infected cells [10, 30], which has also been associated with severe lung lesions after IAV [7, 11, 31]. Our results indicate that early infection of bronchiolar epithelial cells followed by alveolar epithelial cells are the primary source of inflammatory mediators and contribute to the severity of the lung lesion after IAV infection.

Several studies have pointed to the protective role of IFNα release after IAV and other viral infections [32–35]. Here, IFNα was highly upregulated in bronchiolar areas in association with the presence of IAV antigen detected by IHC in bronchiolar epithelial cells. In contrast, the expression of this cytokine in alveolar areas was not as relevant as in the bronchiolar compartment, even though viral replication was extensive in the alveolar compartment at 24 hpi. This differential IFNα expression observed in the lung compartments might reflect diverse intracellular signalling pathways in different cell types, with distinct physiological functions (bronchiolar epithelial cell versus pneumocyte). In that sense, the PRRs TLR3 and RIG-I were also up-regulated differently in the lung compartments studied, which may indicate that the different cell types recognise viral incursions and activate the release of cytokines through different signalling pathways.

In bronchiolar areas, the up-regulation of different immune mediators was associated to a strong up-regulation of RIG-I. RIG-I is activated by the detection of viral RNA in replication and its activation has been associated with protective and enhanced T cell responses against infection and the induction of IFN responses [8, 36].

In contrast to bronchiolar areas, vascular and alveolar areas presented a higher induction of TLR3 but did not show up-regulation of RIG-I at any time point. After IAV infection, TLR3 recognizes infected cells and induces an antiviral state that recruits damage-inducing inflammatory cells contributing to lung pathology [14]. Different studies reported that TLR3 up-regulation was associated with a higher induction of different pro-inflammatory molecules, such as CCL2, particularly in vascular areas [14]. The detrimental role of CCL2 in IAV pathogenesis was proven in a study where CCR2 deficient mice infected with IAV inhibited macrophage migration to the lungs, increasing survival rates [35, 37]. Other studies have also associated CCL2 upregulation with poorer outcomes of infection [10, 11, 38–40]. In our study, vascular areas showed most of CCL2 expression, particularly at 24 hpi, when leukocyte migration was most observed. In addition to CCL2, a higher up-regulation of IL-6 and IFNγ was also observed at 24 hpi in vascular areas, which expression has also been related with severe pathology after IAV infection [10]. These data establish a link between TLR3 signalling and the activation of endothelial and alveolar epithelial cells, leading to the release of different pro-inflammatory mediators, after IAV infection. In the present study, viral active replication was not observed in vascular areas and only low viral antigen was detected by IHC in alveolar areas at early stages of infection, when TLR3 was highly upregulated. TLR3 is expressed in the endosomal compartments of various cellular types, whose activation is mediated by the recognition of double stranded RNA (dsRNA), entered into the cell by endocytosis [20, 21, 41, 42]. Since dsRNA is a universal pathogen-associated molecular pattern (PAMP), it has been assumed that TLR3 would play a key role in antiviral immunity. However, it has been demonstrated that TLR3 also contributes to pulmonary damage through the induction of pro-inflammatory molecules and the recruitment of leukocytes, during IAV virus infection [7, 42–46]. Furthermore, TLR3 polymorphisms have been shown to be related to severe cases of influenza infection in humans and mice [42, 47, 48] and its upregulation has been associated with severe pulmonary lesions in the ferret model after IAV infection [42, 46–49]. Moreover, it is known that TLR3 signalling is not required for the initial cell-autonomous recognition of viral infection or the induction of IFNα, which is induced via viral recognition by RIG-I [50]. These data support the results observed in this study and suggests that viruses actively replicating in the cytoplasm are recognized by RIG-I, but not TLR3, irrespective of their route of entry [50]. Besides, it has been proved that influenza virus-infected cells do not generate dsRNA [51], due to the activity of the cellular RNA helicase UAP56 [52]. Therefore, we hypothesize that TLR3 may recognize unidentified RNA structures, from dying influenza virus-infected cells (cell-derived extracellular RNA or eRNA), or viral components from non-replicative viruses. Supporting this hypothesis, replication-deficient IAV, arisen from internal deletions of IAVs, significantly activated endothelial lung cells inducing a strong host cell response [52]. Besides, it has been recently proved, in vitro and in vivo, that various types of non-self eRNA can be sensed by recipient cells during infection, inducing the release of pro-inflammatory cytokines from recipient cells [17, 53–55].

In summary, our data points to the role of the respiratory bronchiolar epithelium as the first source of pro-inflammatory cytokines and the influx of phagocytic cells into the lungs, early after IAV infection, through the activation of RIG-I. In addition, alveolar and vascular areas contributed to the induction of the pro-inflammatory innate immune response, through the activation of TLR3. In vascular areas, this activation was observed to be independent of active viral replication. We hypothesize that the innate immune response induced by vascular areas, may be consequence of signals triggered by the detection of viral RNA, or, through a complex crosstalk mechanism with the infected lung epithelium.

## Additional files

**Additional file 1.** **Primer sequences.** In this table, primer sequences used for amplification of mRNA by RRT-PCR are shown.

**Additional file 2.** **SELPLG gene expression by RRT-PCR in vascular areas of the lungs.** Comparisons of the gene expression levels of SELPLG in vascular areas of infected and control animals at 12, 24, and 72 hpi. The data expresses the mean with the SEMs. No statistically significant differences were observed between groups.
