# Supplementary material for: Involvement of the different lung compartments in the pathogenesis of pH1N1 influenza virus infection in ferrets
Source: Vet Res. 2016 Nov 8;47:113. doi: 10.1186/s13567-016-0395-0 (PMC5101722; doi:10.1186/s13567-016-0395-0)
Supplement: Supplementary file 1 — Additional file 1. Primer sequences. In this table, primer sequences used for amplification of mRNA by RRT-PCR are shown. [file 13567_2016_395_MOESM1_ESM.doc]

**Additional file 1** Primer sequences.

| **Gene** | | **Primer Sequence (5´- 3´)** | | | **Publication source** |  |
| --- | --- | --- | --- | --- | --- | --- |
| pH1N1  (2009) | M+25  M-124 human09  M+64: | F:AGATGAGTCTTCTAACCGAGGTCG R:TGCAAAGACACTTTCCAGTCTCTG  Probe:FAM^a^-TCAGGCCCCCTCAAAGCCGA-TAMRA^b^ | | | [25] |  |
| IFNα | | F:TCTCCATGTGACAAACCAGAAGA  R:CAGAAAGTCCTGAGCACAATTCC | | | [26] |  |
| IFNγ | | F: TCAAAGTGATGAATGATCTCTCACC  R: GCCGGGAAACACACTGTGAC | | | ” |  |
| TNFα | | F: CCAGATGGCCTCCAACTAATCA  R: GGCTTGTCACTTGGAGTTCGA | | | ” |  |
| IL-6 | | F: AGTGGCTGAAACACGTAACAATTC  R: ATGGCCCTCAGGCTGAACT | | | ” |  |
| IL-8 | | F: AAGCAGGAAAACTGCCAAGAGA  R: GCCAGAAGAAACCTGACCAAAG | | | ” |  |
| TLR3 | | F: GATGACCTCCCAGCAAACAT  R: GCACAATTCTGGCTCCAGTT | | | ” |  |
| CCL2 | | F:GCTCCCTATTCACTTGCTGTTTC  R:GATTCGATAGCCCTCCAGCTT | | | [40] |  |
| β-actin | | | F: GCAGGTCATCACCATCG  R: TGGAGTTGAAGGTGGTCT | [Genbank: AF038150] | | |
| CCL3 | | | F: GGTCTTCTCTGCACCAT  R: CCAGGCTTGGAGCATTG | [Genbank:JP007133] | | |
| RIG-I | | | F: AGAGCACTTGTGGACGCTTT  R: TGCAATGTCAATGCCTTCAT | [Genbank: EU836024.1] | | |
| SELPLG | | | F: CCGCATCCCTGTGAAAC  R: GTGATTCTTGCGGGAGAG | [Genbank:XM4824948.1] | | |
