# Supplementary figures and images for: Involvement of the different lung compartments in the pathogenesis of pH1N1 influenza virus infection in ferrets
Source: Vet Res. 2016 Nov 8;47:113. doi: 10.1186/s13567-016-0395-0 (PMC5101722; doi:10.1186/s13567-016-0395-0)

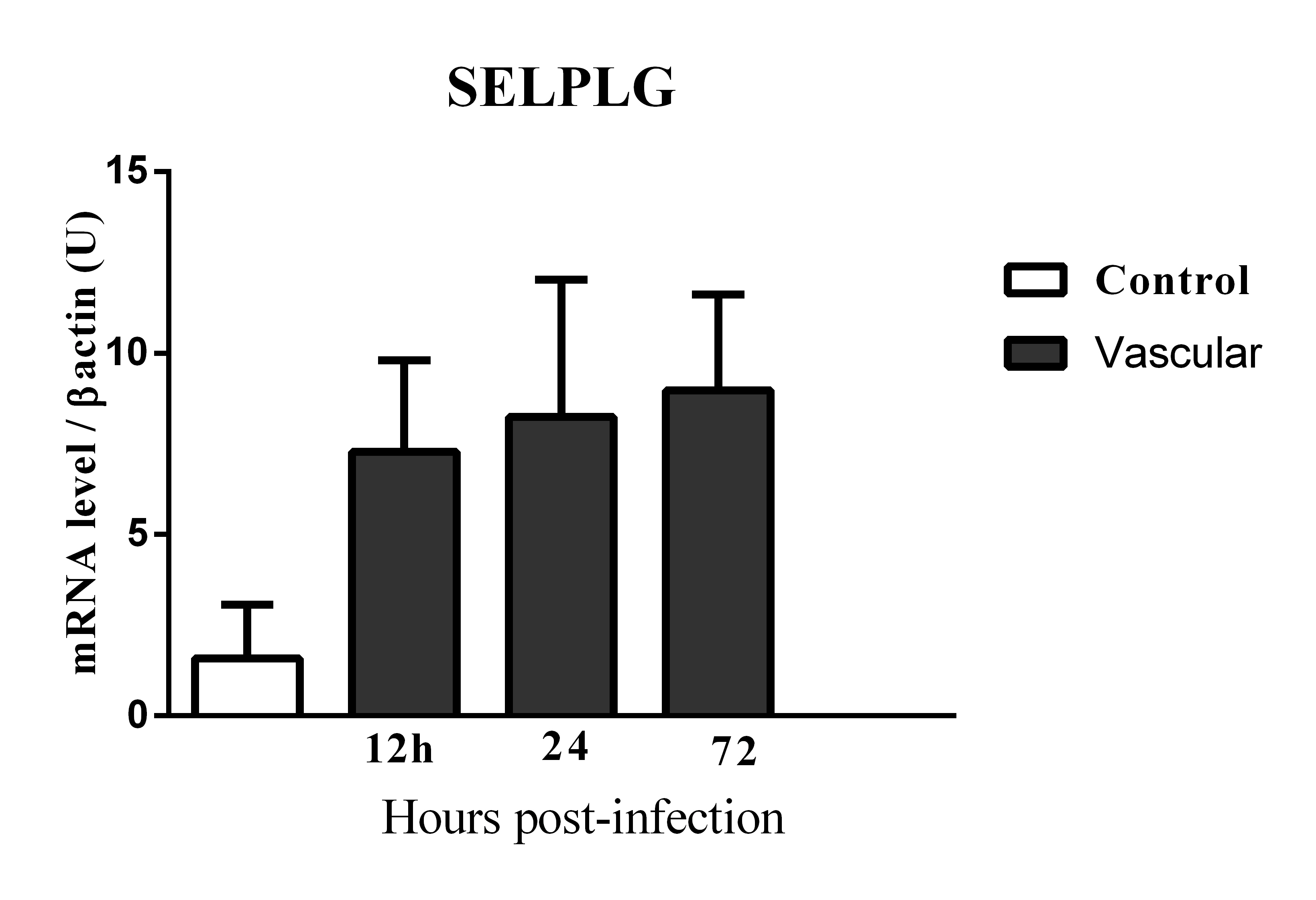

Supplement: Supplementary file 2 — Additional file 2. SELPLG gene expression by RRT-PCR in vascular areas of the lungs. Comparisons of the gene expression levels of SELPLG in vascular areas of infected and control animals at 12, 24, and 72 hpi. The data expresses the mean with the SEMs. No statistically significant differences were observed between groups. [file 13567_2016_395_MOESM2_ESM.jpg]
